# Review of data quality assessment frameworks experiences around Europe

**DOI:** 10.1093/eurpub/ckac129.499

**Published:** 2022-10-25

**Authors:** T Engsig-Karup, P Doupi, M Mäkinen, R Launa, F Estupiñán-Romero, E Bernal-Delgado, N Sahlertz Kristiansen

**Affiliations:** 1 Department of Clinical Epidemiology, Aarhus University Hospital, Aarhus, Denmark; 2 CONNECT, Center for Clinical and Genomic Data, Central Denmark Region, Denmark; 3 Data and Analytics, Finnish Institute for Health and Welfare, Helsinki, Finland; 4 Data Sciences for Health Services and Policy, Institute for Health Sciences in Aragon, Zaragoza, Spain; 5 Health Informatics, Aarhus University Hospital, Aarhus, Denmark

## Abstract

In the first phase of its work, WP6 has explored and synthesized existing knowledge and experiences on data quality assurance frameworks (DQAFs) in the context of cross-border sharing of federated secondary use health data. Our aim was to identify good practices within this area and develop a first set of corresponding recommendations. The recommendations reflect the synthesis of results from three parallel lines of work, each utilizing a different methodology: a) thematic workshops and partner meetings, b) analysis of existing data-sharing initiatives, and c) a scoping literature review. Our analysis of literature and existing health data initiatives suggest that currently deployed DQAFs cannot as such serve as platforms or models for an EHDS-wide data quality solution. There is an agreement in principle, that reliability, relevance, timeliness, coherence, coverage and completeness should be incorporated in a DQAF as measurable dimensions of data quality. We aim towards a working definition of data quality, which will be able to reflect the reality of the data, as well as its fitness for purpose from the perspective of potential users.

• We recommend focusing efforts on transparency at the level of data holder institutions across Member States in relation to adoption of regular audits, a well-developed DQAF and clear procedures with regard to processing the data.

• In the medium to longer-term EHDS nodes could promote and support the development of a benchmarking process, which will assist data managers and data holder institutions with alignment against a Europe-wide approach to measuring data quality within and across Member States.

